# Modulated CH_3_NH_3_PbI_3−x_Br_x_ film for efficient perovskite solar cells exceeding 18%

**DOI:** 10.1038/srep44603

**Published:** 2017-03-17

**Authors:** Yongguang Tu, Jihuai Wu, Zhang Lan, Xin He, Jia Dong, Jinbiao Jia, Panfeng Guo, Jianming Lin, Miaoliang Huang, Yunfang Huang

**Affiliations:** 1 Engineering Research Center of Environment-Friendly Functional Materials for Ministry of Education, Institute of Materials Physical Chemistry, College of Material Science and Engineering, Huaqiao University, Xiamen 361021, China

## Abstract

The organic-inorganic lead halide perovskite layer is a crucial factor for the high performance perovskite solar cell (PSC). We introduce CH_3_NH_3_Br in the precursor solution to prepare CH_3_NH_3_PbI_3−x_Br_x_ hybrid perovskite, and an uniform perovskite layer with improved crystallinity and apparent grain contour is obtained, resulting in the significant improvement of photovoltaic performance of PSCs. The effects of CH_3_NH_3_Br on the perovskite morphology, crystallinity, absorption property, charge carrier dynamics and device characteristics are discussed, and the improvement of open circuit voltage of the device depended on Br doping is confirmed. Based on above, the device based on CH_3_NH_3_PbI_2.86_Br_0.14_ exhibits a champion power conversion efficiency (PCE) of 18.02%. This study represents an efficient method for high-performance perovskite solar cell by modulating CH_3_NH_3_PbI_3−x_Br_x_ film.

Recent years, the solar cells based on organic-inorganic lead halide perovskite are considered as one of the most important developments in the field of solar energy due to their many advantages including low cost, facile preparation process, good stability and high power conversion efficiency (PCE)^1^,^2^,^3^,^4^, meanwhile, organic-inorganic lead halide perovskite unique feature such as broad and strong light absorption^5^, longer carrier lifetimes^6^, long charge carrier diffusion length^7^,^8^, and low exciton binding energy^9^. The perovskite solar cells (PSCs) are generally composed of transparent conducting oxide substrate (TCO), a n-type compact blocking layer, alkylammonium lead halide perovskite layer with or without scaffold layer, a p-type hole-transporting material (HTM) layer and a metal back electrode^1^,^2^. To date, the power conversion efficiency (PCE) of PSC has reached 20.8%^10^. More recently, the highest reported PCE of PSCs has reached 22.1%^11^.

The perovskite layer is a crucial factor for the high performance perovskite solar cells^1^,^2^,^12^,^13^ because perovskite as light harvester absorbs incident irradiation, its energy level dominates the photovoltage and photocurrent of the device, and its morphology affects the charge carrier transportation and the photoelectric properties of the solar cells. Perovskite layers mainly are prepared by sequential deposition^14^, solvent engineering^15^, vapour-assisted deposition^16^, and vacuum evaporation^17^. The most-studied perovskite layers are CH_3_NH_3_PbI_3_^18^, CH_3_NH_3_PbI_3−x_Cl_x_^19^, CH_3_NH_3_PbBr_3_^13^, etc. Noticeably, most high efficiency perovskite solar cells are based on CH_3_NH_3_PbI_3_ or CH_3_NH_3_PbI_3−x_Cl_x_ perovskites^20^. Engineering halide perovskite through mixing halogen elements, such as CH_3_NH_3_PbI_3−x_Cl_x_ and CH_3_NH_3_PbI_3−x_Br_x_, is a viable way to increase the perovskite stability, enhance carrier transport and turn band gap^21^. Recently, CH_3_NH_3_PbI_3−x_Br_x_ based on perovskite solar cells have drawn much attention owing to their variable energy band gaps by adjusting the bromide-iodide ratio^22^. However, the power conversion efficiencies of the perovskite solar cells based on CH_3_NH_3_PbI_3−x_Br_x_ are still lower^21^. As examples, Seok *et al*. reported the meso-structured perovskite solar cells with a PCE of 12.3% by utilizing the traditional one-step spin-coating method for CH_3_NH_3_PbI_3−x_Br_x_ deposition^22^. Huang *et al*. achieved a PCE of 13.1% in CH_3_NH_3_PbI_3−x_Br_x_-based planar perovskite solar cells by using the two-step spin-coating method with the solvent annealing process^23^. Yuan *et al*. achieved a high fill factor exceeding 85% and a power conversion efficiency exceeding 13% in CH_3_NH_3_PbI_3−x_Br_x_ based perovskite solar cells^20^.

Herein, we introduce CH_3_NH_3_Br in the precursor solution to prepare mixed methylammonium lead halide CH_3_NH_3_PbI_3−x_Br_x_, which results in the significant improvement of photovoltaic performance of PSCs. Firstly, uniform perovskite layer with improved crystallinity and apparent grain contour is obtained, less grain boundaries will facilitate the transmission of charge. Secondly, owing to the introduction of Br into CH_3_NH_3_PbI_3_, the energy band of perovskite is expanded and leads to a higher photovoltage (*V*_*OC*_) of the device. Thirdly, the modulation of Br is beneficial to the interfacial energy match between TiO_2_/perovskite/spiro-OMeTAD, the injection and extraction of charge carriers are improved and thus the photocurrent and fill factor of the device are enhanced. Under optimal conditions, the device with CH_3_NH_3_PbI_2.86_Br_0.14_ exhibits high reproducibility and a champion PCE of 18.02%.

## Results

### Crystallinity and morphology

**Figure 1:**
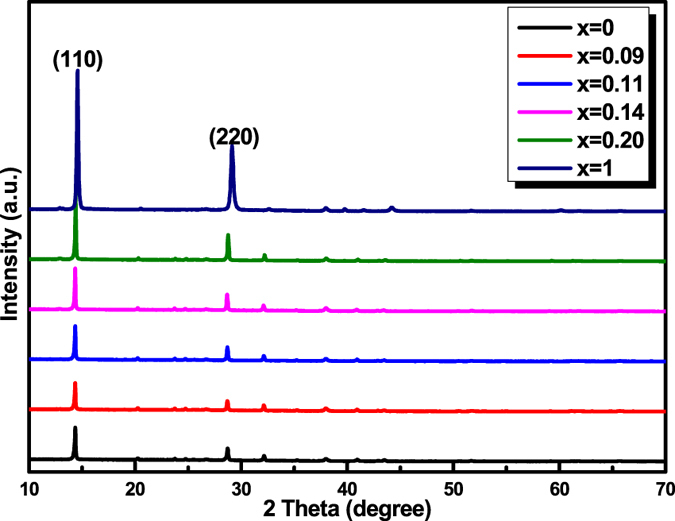
XRD patterns of CH_3_NH_3_PbI_3−x_Br_x_ perovskite films.

CH_3_NH_3_PbI_3−x_Br_x_ perovskites were prepared by controlling different iodide/bromide molar ratio in perovskite precursor solutions. The resultant mixed methylammonium lead halides are termed as CH_3_NH_3_PbI_3_, CH_3_NH_3_PbI_2.91_Br_0.09_, CH_3_NH_3_PbI_2.89_Br_0.11_, CH_3_NH_3_PbI_2.86_Br_0.14_, CH_3_NH_3_PbI_2.8_Br_0.2_, and CH_3_NH_3_PbI_2_Br, respectively. Figure 1 shows the XRD patterns of the CH_3_NH_3_PbI_3−x_Br_x_ perovskite films. The two main peaks for all CH_3_NH_3_PbI_3−x_Br_x_ films approximately located at 14.2° and 28.4° can be indexed to the (110) and (220) planes^24^,^25^, confirming the presence of the tetragonal perovskite phase in all of the prepared films. In order to examine the influence of Br amount on the crystallinity of the perovskite films, XRD patterns of CH_3_NH_3_PbI_3−x_Br_x_ films at 13.5°~15.5° were measured and shown in Figure S1. The FWHM values of the strong diffraction peaks of the (110) face are calculated using JADE 6.0^26^,^27^. The FWHM values of the CH_3_NH_3_PbI_3−x_Br_x_ films are 0.17°, 0.18°, 0.16°, 0.14°, 0.13° and 0.12°, corresponding to the CH_3_NH_3_PbI_3−x_Br_x_, x = 0, 0.09, 0.11, 0.14, 0.20, and 1, respectively. Small FWHM value means a sharper XRD pattern^28^,^29^. On the other hand, the characteristic (110) peak has shifted from CH_3_NH_3_PbI_3_ at 14.30° to CH_3_NH_3_PbI_2_Br at 14.09°, corresponding to a reduction in the lattice parameter from a = 0.616 nm to a = 0.604 nm with the increase in the Br content due to the difference in the ionic radius of Br^−^ (1.96 A) and I^−^ (2.2 A), which is consistent with the literatures^22^,^30^.

**Figure 2:**
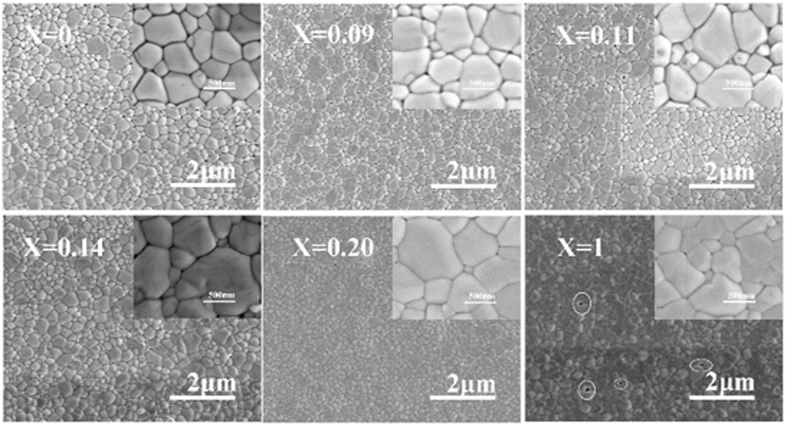
Top-view SEM images of CH_3_NH_3_PbI_3−x_Br_x_ perovskite films.

Top-view SEM images of as-prepared CH_3_NH_3_PbI_3−x_Br_x_ hybrid perovskite films are shown in Fig. 2. It can be seen that the Br amount has a mild influence on the grain size of the hybrid perovskite. CH_3_NH_3_PbI_2.86_Br_0.14_ and CH_3_NH_3_PbI_2.8_Br_0.2_ present compact, pinhole-free perovskite films and enlarged average grain size, which will minimize the grain boundary energy and be beneficial to the charge transportation. For CH_3_NH_3_PbI_2_Br, there are some pinholes appearing in the film, marked by white circles. The pinholes will produce direct contacts of spiro-OMeTAD and electron transporting layer, leading to a shunting path which probably decreases fill factor and open-circuit voltage for the device with CH_3_NH_3_PbI_2_Br^17^.

**Figure 3:**
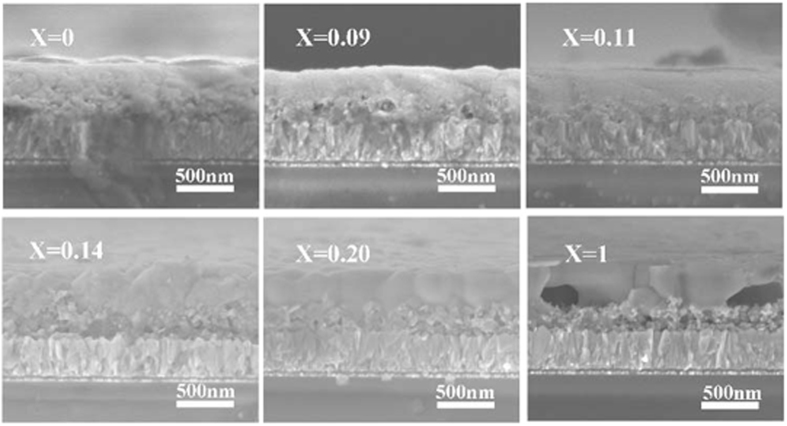
Cross-view SEM images of CH_3_NH_3_PbI_3−x_Br_x_ perovskite films.

Figure 3 shows the cross-view SEM images as-prepared CH_3_NH_3_PbI_3−x_Br_x_ perovskite films. Surprisingly, there are some stark contrasts of the perovskite film in vertical direction. The cementite appears as small granular grain in the capping perovskite layer for CH_3_NH_3_PbI_3_ and CH_3_NH_3_PbI_2.91_Br_0.09_. With the increase of Br amount from CH_3_NH_3_PbI_3_ to CH_3_NH_3_PbI_2_Br, the big grain contour becomes gradually obvious. The grain size is commensurate with its thickness among CH_3_NH_3_PbI_2.86_Br_0.14_, CH_3_NH_3_PbI_2.8_Br_0.2_ and CH_3_NH_3_PbI_2_Br, most of the grain boundaries are perpendicular to the mesoporous layer to minimize the grain boundaries. As well known that the less grain boundaries will facilitate the transportation of charge^29^,^31^, thus it is expected that the fill factor of the corresponding device is improved by adjusting Br amount in CH_3_NH_3_PbI_3−x_Br_x_ hybrid perovskite.

However, for CH_3_NH_3_PbI_2_Br, there are many voids at the bottom of the perovskite layer which may be attributed to the dewetting during anti-solvent washing and subsequent growth^29^,^31^,^32^. In the specific experimental operation of CH_3_NH_3_PbI_2_Br, the viscosity of the precursor solution may vary owing to different compositions in the precursor solution and the methylammonium halide was composed entirely of MABr. As the nucleation grows the material dewets at the interface between solution-phase and solid-phase, meaning that it is pulled away from the regions surrounding the crystals, resulting in the formation of large voids in the film. So a distinguishing crystallization behavior was obtained without MAI in the precursor solution. Many voids in the perovskite layer will result in inferior photovoltaic performance of PSCs.

### Band gap

**Figure 4:**
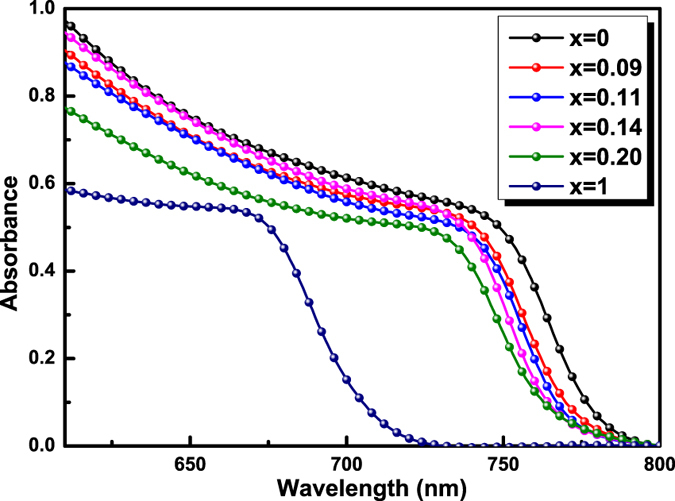
UV-visible absorption spectra of CH_3_NH_3_PbI_3−x_Br_x_ perovskite films.

Figure 4 shows the UV-visible absorption spectra of the CH_3_NH_3_PbI_3−x_Br_x_ perovskite films with different iodide/bromide ratio. It can be seen that with the increase of Br amount from CH_3_NH_3_PbI_3_ to CH_3_NH_3_PbI_2_Br, the absorption edges of the hybrid perovskite films shift towards the shorter wavelength^33^. The bandgaps of crystallized perovskite films are 1.565, 1.579, 1.585, 1.591, 1.597, and 1.725 eV, corresponding to the CH_3_NH_3_PbI_3−x_Br_x_, x = 0, 0.09, 0.11, 0.14, 0.20, and 1, respectively, from the Taus plots^34^ shown in Figure S2. The corresponding absorption edge and band gap are listed in Table 1. The blue shift is found that: 782 nm for CH_3_NH_3_PbI_3_ is shifted to 709 nm for CH_3_NH_3_PbI_2_Br, consistent with the variety of the energy band levels of perovskite materials in Figure S3. According to the literatures^34^,^35^,^36^ and the energy level principle^2^,^3^,^12^,^37^,^38^, with the increase of Br amount in CH_3_NH_3_PbI_3−x_Br_x_ the conduction band of the perovskite ascends and the valence band descends, resulting in the absorption edge shifted towards to shorter wavelength, which indicates the formation of wider energy band and a higher photovoltage (*V*_*OC*_) of the device.

### Charge carrier lifetime

**Figure 5:**
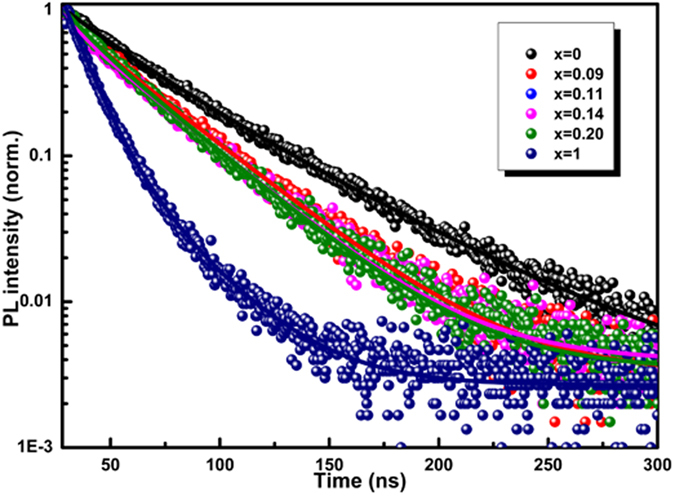
Time-resolved photoluminescence intensity decay of CH_3_NH_3_PbI_3−x_Br_x_ detected at peak emission wavelength of 760 nm.

Time resolved photoluminescence (TRPL) intensity decay measurements of CH_3_NH_3_PbI_3−x_Br_x_ perovskite films offer a quantitative information on the dynamics of charge carrier recombination^39^, the samples were composed of FTO/CL-TiO_2_/mp-TiO_2_/perovskite, and corresponding results are presented in Fig. 5, of which the fitted time constants charge carrier lifetime (τ) are 52, 39, 35, 34, 33, and 15 ns, corresponding to the CH_3_NH_3_PbI_3−x_Br_x_, x = 0, 0.09, 0.11, 0.14, 0.20, and 1, respectively. The shorter τ value means that the charge more fast transfers from the perovskite to TiO_2_ layer^40^,^41^,^42^,^43^,^44^. The significantly reduced τ value with the increase of Br amount in the hybrid perovskite infers an efficient charge extraction process occurred at the perovskite/TiO_2_ interface. The CH_3_NH_3_PbI_2_Br yields the smallest τ value, meaning that the charge carriers extract very quickly, which may be attributed to the high conduction band of the perovskite. The high conduction band energy level is beneficial to the charge injection between the perovskite and the electron selective electrode, however, the device of CH_3_NH_3_PbI_2_Br presents the worst performance.

### Charge transferring resistances

**Figure 6:**
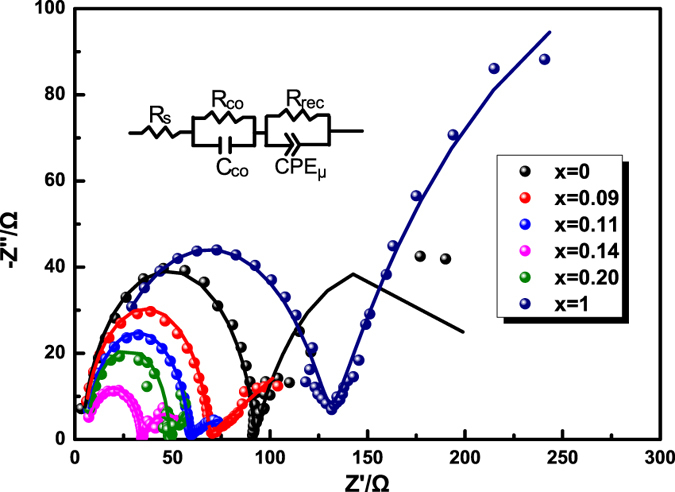
Nyquist plots of CH_3_NH_3_PbI_3−x_Br_x_ films.

Nyquist plots of CH_3_NH_3_PbI_3−x_Br_x_ films are shown in Fig. 6, and the corresponding equivalent circuit is inset. The radius of semi-circles corresponds to the interfacial charge transfers resistance^45^,^46^. It can be seen that with the increase of X (Br amount), the radii first decrease, and then increase. When X = 0.14, the radius reaches the minimum, indicating the smallest interfacial charge transfers resistance among all CH_3_NH_3_PbI_3−x_Br_x_ films. Therefore, the device based on CH_3_NH_3_PbI_2.86_Br_0.14_ film possesses superior charge injection characteristics and low internal resistance, resulting in better photovoltaic performance than others.

### Photovoltaic performance

**Figure 7:**
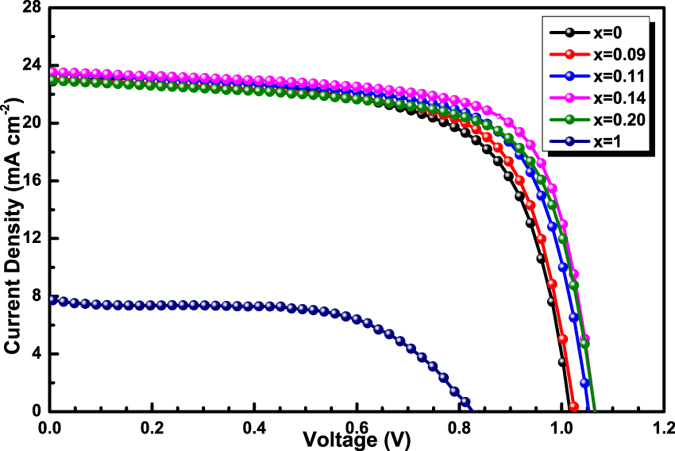
*J-V* curves of the PSCs based on CH_3_NH_3_PbI_3−x_Br_x_ under AM 1.5 G illumination.

*J-V* curves (reverse scan) of the perovskite solar cells based on CH_3_NH_3_PbI_3−x_Br_x_ hybrid perovskite are shown in Fig. 7 and the corresponding photovoltaic parameters are listed in Table 1. For the device based on ancestral CH_3_NH_3_PbI_3_ film, a PCE of 15.60% is obtained with short-circuit current density (*J*_*SC*_) of 22.92 mA·cm^*–*2^, open-circuit photovoltage (*V*_*OC*_) of 1.016 V and fill factor (*FF*) of 0.67. For the devices based on CH_3_NH_3_PbI_3−x_Br_x_ films (x = 0~0.14), the performance continuously improve with the increase of Br amount. Best performance is achieved in the PSC based on CH_3_NH_3_PbI_2.86_Br_0.14_, yielding a *J*_*SC*_ of 23.52 mA·cm^−2^, *V*_*OC*_ of 1.064 V, and *FF* of 0.72, resulting in a PCE of 18.02%. Further increasing the amount of Br in CH_3_NH_3_PbI_2.8_Br_0.2_ do not improve device performance, resulting from a lower *J*_*SC*_ of 22.95 mA·cm^−2^. Furthermore, the performance of the device based on CH_3_NH_3_PbI_2_Br shows a dramatic downward.

The reason for the increase and then decrease of power conversion efficiency (PCE) of the PSCs with the increase of Br amount lies in *V*_*OC*_, *J*_*SC*_ and *FF*. From the aspect of PV energy devices^3^,^17^,^37^,^38^, with the increase of Br amount, the conduction band of the perovskite ascends and the valence band descends, the energy difference (ΔE_1_) between TiO_2_ Femi level and the valence band of perovskite expends, the energy difference (ΔE_2_) between the conduction band of perovskite and TiO_2_ Femi level also expends. The expanded ΔE_1_ increases *V*_*OC*_, however appropriate ΔE_2_ produces small energy loss and large *J*_*SC*_ and FF; and excess ΔE_2_ by excessive Br leads to an opposite result. From the aspect of morphology, owing to a large amount of voids in CH_3_NH_3_PbI_2_Br perovskite by dewetting, the charge extraction process is limited at the interface of perovskite/TiO_2_. Combined above factors, photovoltaic parameters of the PSCs based on CH_3_NH_3_PbI_3−x_Br_x_ increase and then decrease of with the increase of Br amount.

Noticeably, owing to different preparation conditions, materials and devices structure, the optimized bromide/iodide ratio may be different. For example, Jeon *et al*. reported an optimal perovskite CH_3_NH_3_PbI_3−x_Br_x_ (x = 0.3~0.45)^15^, and He *et al*. obtained an efficiency of 12.1% in planar heterojunction device by using CH_3_NH_3_PbI_2.4_Br_0.6_^47^.

Figure S4 and Table S1 show the *J-V* curves and the photovoltaic parameters of the devices based on CH_3_NH_3_PbI_3−x_Br_x_ films (x = 0, 0.09, 0.14, and 1) under both reverse and forward bias scans. It can be seen that there are still noticeable hysteresis effects and instabilities for all samples. However, the device based on the champion film CH_3_NH_3_PbI_2.86_Br_0.14_ shows the highest reverse PCE (18.02%) and forward PCE (14.62%).

**Figure 8:**
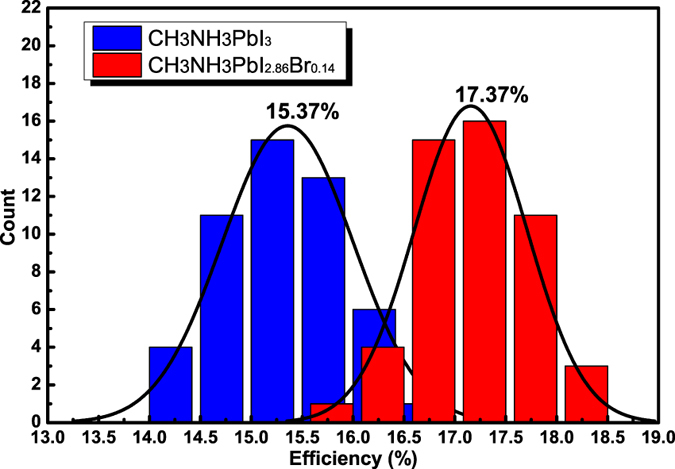
Histograms of the PCEs of the PSCs with CH_3_NH_3_PbI_3_ and CH_3_NH_3_PbI_2.86_Br_0.14_.

Statistic results of the cell performance are provided in Fig. 8 as histogram charts. It can be found that the devices with CH_3_NH_3_PbI_2.86_Br_0.14_ shows better performance and the average PCE is 17.37%. Meanwhile, the histogram chart demonstrates the high reproducibility of the devices (Each team is calculated from a batch of 50 cells). The corresponding incident-photon-to-current conversion efficiency (IPCE) spectra of PSC devices are shown in Figure S5.

*J-V* curves of the devices based on CH_3_NH_3_PbI_2.86_Br_0.14_ with various dwell time from 30 ms to 800 ms are shown in Figure S6, it can be seen that there are minor fluctuations. Figure S7 shows the steady-state photocurrent and output PCE of the device based on CH_3_NH_3_PbI_2.86_Br_0.14_ at the maximum power points with a stabilized current density output of 20.10 mA·cm^−2^ (at the voltage of 0.88 V), yielding a PCE of 17.68%.

## Discussions

In summary, we introduce CH_3_NH_3_Br in perovskite precursor solution to prepare CH_3_NH_3_PbI_3−x_Br_x_, leading to the significant improvement of photovoltaic performance of perovskite solar cell. Firstly, uniform perovskite layer with improved crystallinity and apparent grain contour is obtained, and less grain boundaries will facilitate the transportation of charges. Secondly, owing to the introduction of Br, the energy band of perovskite is expanded, resulting in a higher photovoltage of the device. Thirdly, the appropriate regulation of energy level by Br is beneficial to interfacial energy match and the charge injection and extraction at between TiO_2_/perovskite/spiro-OMeTAD and enhances photocurrent, which is certified by time-resolved photoluminescence intensity decay measurements. Under optimal conditions, the device based on CH_3_NH_3_PbI_2.86_Br_0.14_ achieves a champion PCE of 18.02% with a stabilized output efficiency of 17.68% at the maximum power point.

## Methods

### Materials and Reagents

All of the materials were purchased from the Sigma-Aldrich Corp., if not specified. The Spiro-OMeTAD was from Luminescence Technology Corp. CH_3_NH_3_I was synthesized according to literature^48^.

### Precursor solution preparation

461 mg PbI_2_ (99.9985%, Alpha Aesar) and 78 mg DMSO were mixed in 702 μL DMF solution at room temperature. Then, mixed methylammonium halides (MAX, X = I, Br) were added to the above solution, the mole ratio of PbI_2_: MAX: DMSO was controlled at 1:1:1. The molar ratio of MAI: MABr in MAX was controlled at: 1:0, 10:1, 8:1, 6:1, 4:1, 0:1, respectively. Lastly, the solutions were stirred at room temperature for 1 h in order to form a completely dissolved perovskite precursor solution. The resultant perovskites were termed as CH_3_NH_3_PbI_3_, CH_3_NH_3_PbI_2.91_Br_0.09_, CH_3_NH_3_PbI_2.89_Br_0.11_, CH_3_NH_3_PbI_2.86_Br_0.14_, CH_3_NH_3_PbI_2.8_Br_0.2_, and CH_3_NH_3_PbI_2_Br, respectively.

### Device fabrication

Fluorine-doped tin oxide-coated (FTO) glass (Pilkington, TEC-8, 8Ω/sq) was cleaned by UV-ozone treatment for 15 min, followed by cleaning with detergent and ethanol consecutively. The compact TiO_2_ blocking layers were deposited on FTO glass, which was prepared by spin-coating 0.15 M titanium diisopropoxide bis(acetylacetonate) (75 wt% in isopropanol, Aldrich) in 1-butanol (99.8%, Aldrich) solution, at 500 rpm for 5 s and 2000 rpm for 30 s and then dried at 125 °C for 5 min. A mesoporous TiO_2_ film was deposited on compact TiO_2_ layer by spin-coating TiO_2_ paste diluted in ethanol. TiO_2_ paste was prepared as described previously^49^,^50^,^51^. After drying at 100 °C for 5 min, the film was annealed at 450 °C for 30 min, providing a thickness of *ca.* 200 nm. The mesoporous TiO_2_ film was immersed in 0.02 M aqueous TiCl_4_ solution at 80 °C for 20 min. After washing with deionized water and alcohol, the film was heated at 500 °C for 30 min. 20 μL precursor solution was dipped onto the mesoporous TiO_2_ layer and then spun at 4000 rpm for 25 sec. During spinning, 0.5 mL of diethyl ether was slowly dripped on the rotating substrate within the first 7 s. The film was finally heated to 60 °C for about 2 min and followed by 100 °C for 10 min.

A volume of 20 μL of 2,2′,7,7′-tetrakis (N,N-di-p-methoxyphenylamine) −9,9-spirobifluorene (Spiro-OMeTAD) solution was spin-coated on the CH_3_NH_3_PbI_3_ perovskite layer at 4,000 rpm for 30 s. A Spiro-OMeTAD solution was prepared by dissolving 72.3 mg of Spiro-OMeTAD in 1 mL of chlorobenzene, to which 28.8 μL of 4-tert-butyl pyridine and 17.5 μL of lithium bis(trifluoromethanesulfonyl)imide (Li-TFSI) solution (520 mg LI-TSFI in 1 mL acetonitrile, Sigma-Aldrich, 99.8%) were added. All devices were stored in a desiccator (humidity < 15%) in the dark for 12 h. Finally, 80 nm of gold was deposited under vacuum through a shadow mask.

### Characterization

The current density-voltage *(J-V)* curves were measured using a Keithley 2420 source-measure unit under AM1.5 G illumination at 100 mW·cm^−2^ provided by an Oriel Sol 3 A solar simulator in ambient environment. The light intensity was adjusted using a NREL-calibrated Si solar cell equipped with KG-2 filter. The devices had an active area of 0.125 cm^2^ without metal mask. The devices were measured by reverse (2.0 to −0.1 V) and forward (−0.1 to 2.0 V) voltage scanning at a scan step of about 21.2 mV (100 data points in total). The pre-sweep delay time was 40 ms, the dwell time at each voltage step was 30 ms. Surface morphologies were characterized by field-emission scanning electron microscopy (SEM, Hitachi S-8000, Japan). The crystalline structures were examined using X-ray diffraction (XRD, Bruker AXS, D8 Advance). Impedance spectra (IS) for the solar cell were measured on a ZAHNER IM6e electrochemical workstation under 1 sun AM 1.5 illumination, by applying a 0 V DC bias and a 5 mV voltage perturbation in the frequency range from 0.1 to 10^6^ Hz. The impedance spectra were analyzed with Zview software. *IPCE* curves were measured as a function of wavelength from 300 nm to 800 nm using the Newport IPCE system (Newport, USA). The time-resolved photoluminescence spectrum was acquired using the time-correlated single-photon counting technique (Pico harp 300), and the excitation light pulse was provided using a picosecond diode laser at a wavelength of 760 nm with a repetition frequency of 1 MHz (PDL 800B).

## Additional Information

**How to cite this article:** Tu, Y. *et al*. Modulated CH_3_NH_3_PbI_3−x_Br_x_ film for efficient perovskite solar cells exceeding 18%. *Sci. Rep.* **7**, 44603; doi: 10.1038/srep44603 (2017).

**Publisher's note:** Springer Nature remains neutral with regard to jurisdictional claims in published maps and institutional affiliations.

## Supplementary Material

Supplementary Information

## Figures and Tables

**Table 1:** Energy data and photovoltaic parameters of PSCs with CH_3_NH_3_PbI_3−x_Br_x_.

CH_3_NH_3_PbI_3−x_Br_x_	Absorption edge (nm)	Band gap (eV)	V_OC_ (V)	J_SC_ (mA·cm^–2^)	FF	PCE(%)
x = 0	782	1.565	1.016	22.92	0.67	15.60
x = 0.09	775	1.579	1.026	23.04	0.69	16.31
x = 0.11	772	1.585	1.052	23.08	0.69	16.75
x = 0.14	769	1.591	1.064	23.52	0.72	18.02
x = 0.20	766	1.597	1.065	22.95	0.70	17.11
x = 1	709	1.725	0.827	7.72	0.60	3.83
