# Supplementary material for: Modulated CH3NH3PbI3−xBrx film for efficient perovskite solar cells exceeding 18%
Source: Sci Rep. 2017 Mar 17;7:44603. doi: 10.1038/srep44603 (PMC5355988; doi:10.1038/srep44603)
Supplement: Supplementary Information [file srep44603-s1.doc]

**Supporting Information**

**for Scientific Reports, DOI: 10.1038/srep (XXXX).**

**Modulated CH_3_NH_3_PbI_3-x_Br_x_ film for efficient perovskite solar cells exceeding 18 %**

Yongguang Tu, Jihuai Wu[[1]](#footnote-2), Zhang Lan, Xin He, Jia Dong, Jinbiao Jia, Panfeng Guo, Jianming Lin, Miaoliang Huang, Yunfang Huang

*Engineering Research Center of Environment-Friendly Functional Materials for Ministry of Education, Institute of Materials Physical Chemistry, College of Material Science and Engineering, Huaqiao University, Xiamen 361021, China*

**Figures S1-S7 and Table S1**

**Figure S1**. XRD patterns of CH_3_NH_3_PbI_3-x_Br_x_ perovskite films.

**Figure S2.** Tauc Plots of the as-prepared perovskite films.


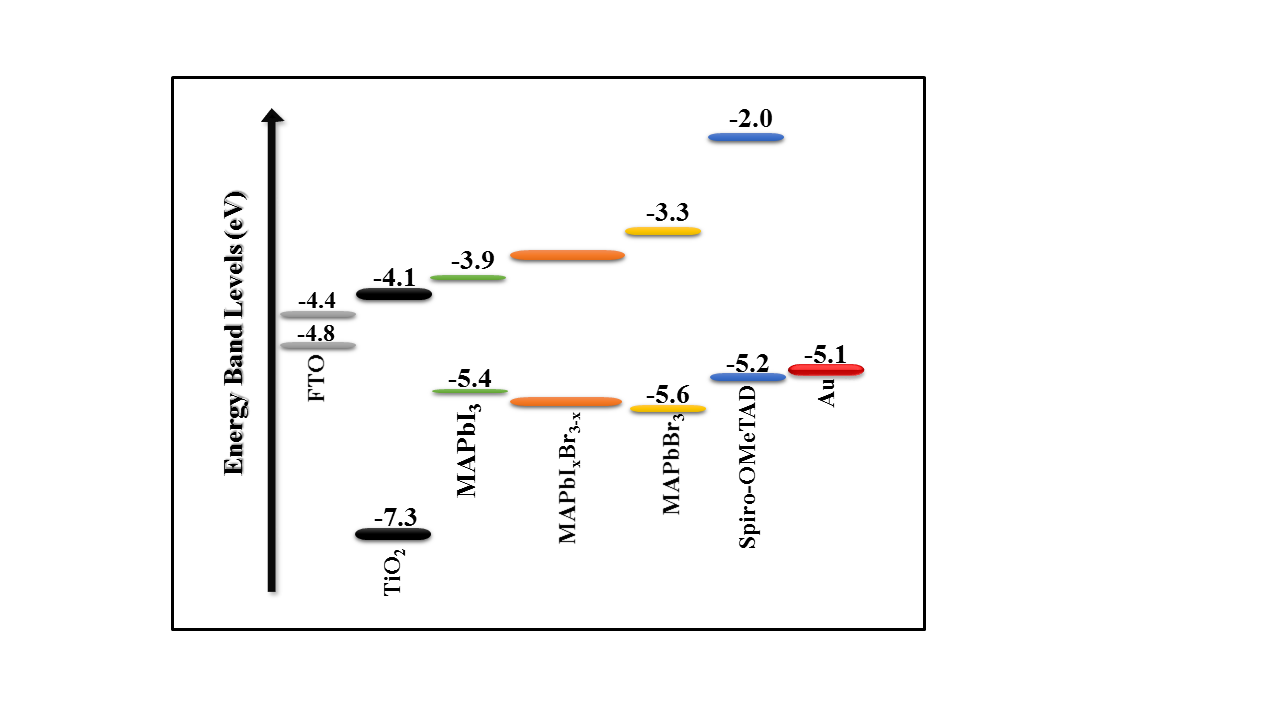


**Figure S3****.** The energy bands of materials frequently used in perovskite solar cells.

**Figure S4.** *J-V* curves of the perovskite solar cell based on IPA substitution for 60 s measured by reverse (open circuit → short circuit) and forward (short circuit → open circuit) scans under one sun illumination.

**Figure S5.** *IPCE* spectra of the perovskite solar cells based on CH_3_NH_3_PbI_3-x_Br_x_.

**Figure S6.** *J–V* curves of the PSCs based on CH_3_NH_3_PbI_2.86_Br_0.14_ with various dwell time (30 to 800 ms). Scans from forward bias to short-circuit.

**Figure S7.** The steady-state photocurrents and output PCE of the devices at the maximum power points.

**Table S1**. Photovoltaic parameters of PSCs with CH_3_NH_3_PbI_3-x_Br_x_ measured by reverse and forward scans under one sun illumination.

| CH_3_NH_3_PbI_3-x_Br_x_ | Scan direction | V_OC_(V) | J_SC_(mA cm^–2^) | FF | PCE(%) |
| --- | --- | --- | --- | --- | --- |
| X=0 | Reverse | 1.016 | 22.92 | 0.67 | 15.60 |
|  | Forward | 1.018 | 22.92 | 0.54 | 12.59 |
| X=0.09 | Reverse | 1.026 | 23.04 | 0.69 | 16.31 |
|  | Forward | 1.028 | 23.05 | 0.58 | 13.74 |
| X=0.14 | Reverse | 1.064 | 23.52 | 0.72 | 18.02 |
|  | Forward | 1.019 | 23.52 | 0.61 | 14.62 |
| X=1 | Reverse | 0.827 | 7.72 | 0.60 | 3.83 |
|  | Forward | 0.761 | 7.72 | 0.41 | 2.41 |

1. Corresponding author. Tel: +86-595-22693899; Fax: +86-595-22692229; E-mail address: jhwu@hqu.edu.cn (J. Wu). [↑](#footnote-ref-2)
